# XYZeq: Spatially resolved single-cell RNA sequencing reveals expression heterogeneity in the tumor microenvironment

**DOI:** 10.1126/sciadv.abg4755

**Published:** 2021-04-21

**Authors:** Youjin Lee, Derek Bogdanoff, Yutong Wang, George C. Hartoularos, Jonathan M. Woo, Cody T. Mowery, Hunter M. Nisonoff, David S. Lee, Yang Sun, James Lee, Sadaf Mehdizadeh, Joshua Cantlon, Eric Shifrut, David N. Ngyuen, Theodore L. Roth, Yun S. Song, Alexander Marson, Eric D. Chow, Chun Jimmie Ye

**Affiliations:** 1 Department of Microbiology and Immunology, University of California, San Francisco, San Francisco, CA 94143, USA.; 2 Diabetes Center, University of California, San Francisco, San Francisco, CA 94143, USA.; 3 Innovative Genomics Institute, University of California, Berkeley, Berkeley, CA 94720, USA.; 4 J. David Gladstone Institutes, San Francisco, CA 94158, USA.; 5 Department of Biochemistry and Biophysics, University of California, San Francisco, San Francisco, CA 94158, USA.; 6 Center for Advanced Technology, University of California, San Francisco, San Francisco, CA 94158, USA.; 7 Graduate Group in Biostatistics, University of California, Berkeley, CA 94720, USA.; 8 Center for Computational Biology, University of California, Berkeley, CA 94720, USA.; 9 Graduate Program in Biological and Medical Informatics, University of California, San Francisco, San Francisco, CA 94158, USA.; 10 Medical Scientist Training Program, University of California, San Francisco, San Francisco, CA 94143, USA.; 11 Biomedical Sciences Graduate Program, University of California, San Francisco, San Francisco, CA 94143, USA.; 12 Division of Rheumatology, Department of Medicine, University of California, San Francisco, CA 94143, USA.; 13 Division of Hematology and Oncology, University of California, San Francisco, San Francisco, CA 94143, USA.; 14 Scienion AG, Volmerstrasse 7b, 12489 Berlin, Germany.; 15 Department of Medicine, University of California, San Francisco, San Francisco, CA 94143, USA.; 16 Computer Science Division, University of California, Berkeley, CA 94720, USA.; 17 Department of Statistics, University of California, Berkeley, CA 94720, USA.; 18 Chan Zuckerberg Biohub, San Francisco, CA 94158, USA.; 19 UCSF Helen Diller Family Comprehensive Cancer Center, University of California, San Francisco, San Francisco, CA 94158, USA.; 20 Parker Institute for Cancer Immunotherapy, University of California, San Francisco, San Francisco, CA 94129, USA.; 21 Institute for Human Genetics, University of California, San Francisco, San Francisco, CA 94143, USA.; 22 Gladstone–UCSF Institute of Genomic Immunology, San Francisco, CA 94158, USA.; 23 Institute of Computational Health Sciences, University of California, San Francisco, San Francisco, CA 94143, USA.; 24 Department of Epidemiology and Biostatistics, University of California, San Francisco, San Francisco, CA 94158, USA.

## Abstract

Single-cell RNA sequencing (scRNA-seq) of tissues has revealed remarkable heterogeneity of cell types and states but does not provide information on the spatial organization of cells. To better understand how individual cells function within an anatomical space, we developed XYZeq, a workflow that encodes spatial metadata into scRNA-seq libraries. We used XYZeq to profile mouse tumor models to capture spatially barcoded transcriptomes from tens of thousands of cells. Analyses of these data revealed the spatial distribution of distinct cell types and a cell migration-associated transcriptomic program in tumor-associated mesenchymal stem cells (MSCs). Furthermore, we identify localized expression of tumor suppressor genes by MSCs that vary with proximity to the tumor core. We demonstrate that XYZeq can be used to map the transcriptome and spatial localization of individual cells in situ to reveal how cell composition and cell states can be affected by location within complex pathological tissue.

## Teaser

XYZeq is a novel scalable platform that directly encodes spatial location from tissue into single-cell RNA sequencing libraries.

## INTRODUCTION

Over the past decade, massively parallel single-cell RNA sequencing (scRNA-seq) has emerged as a powerful approach to catalog the remarkable cellular heterogeneity in complex tissues (*1*, *2*). While scRNA-seq can profile the transcriptomes of thousands of cells in a single experiment, it requires the dissociation of tissue into single-cell suspensions before library preparation and sequencing, eliminating any spatial information (*3*–*6*). Several strategies have emerged to obtain molecular and spatial information simultaneously from complex tissue. Imaging-based strategy combines high-resolution microscopy with fluorescence in situ hybridization to achieve subcellular resolution and could profile the entire transcriptome (*7*–*10*), but this requires lengthy iterative microscopy workflows and large probe panels. Another approach is to hybridize RNA directly from tissue slices onto a microarray containing spatially barcoded oligo(dT) spots or beads to encode location information into RNA-seq libraries. These approaches can sample the entire transcriptome without the need for iterative rounds of hybridization (*11*), and recent improvements using DNA-barcoded beads (high-definition spatial transcriptomics and Slide-seqv1/v2) report spatial resolutions at or below the diameter of a single cell (*12*–*14*). However, because of the low numbers of mRNA molecules captured per bead, these spatial transcriptomic approaches often aggregate neighboring beads before downstream analysis, resulting in lower effective resolution and averaging of transcript abundances from multiple cells. As a result, annotation of specific cell types present within each spatial unit of analysis is accomplished by aggregating gene sets computationally defined from orthogonal scRNA-seq datasets (*15*, *16*). While integration methods have demonstrated the ability to localize cell types within the spatial organization of complex tissue, they rely on having available data from two independent assays and have limited ability to infer how spatial context influences the cell state of individual cell types.

## RESULTS

**Fig. 1:**
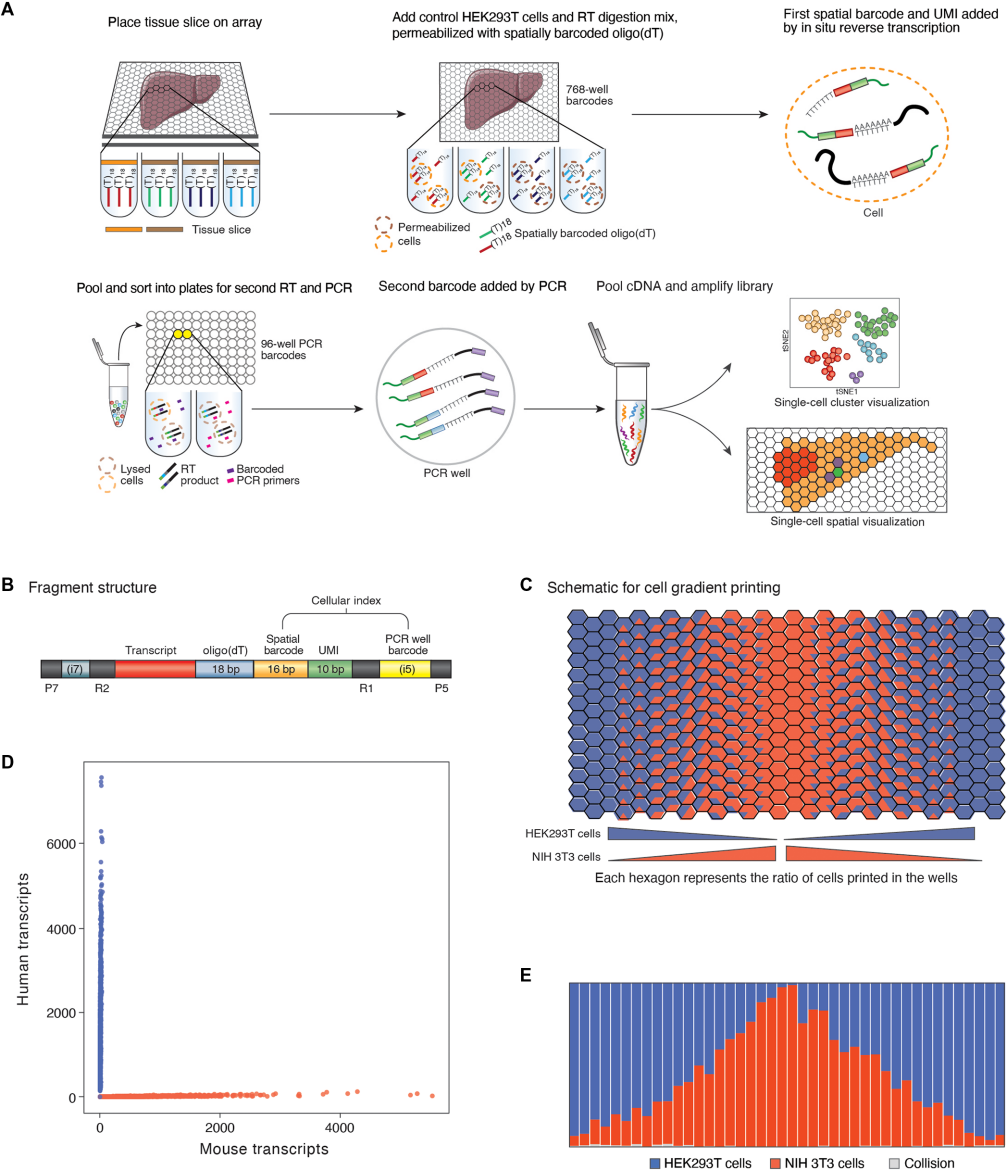
XYZeq enables single-cell and spatial transcriptome profiling simultaneously. (**A**) Schematic of the XYZeq workflow. (**B**) Schematic of XYZeq sequencing library structure. P5 and P7, Illumina adaptors; bp, base pairs; R1 and R2, annealing sites for Illumina sequencing primers. (**C**) Schematic representation of the mixed-species cell gradient pattern printed on the chip with 11 unique cell proportion ratios (see Methods for specific cell proportion ratios). (**D**) Scatterplot of mouse (*x* axis) and human (*y* axis) UMI counts detected from a mixture of HEK293T and NIH 3T3 cells after computational decontamination. Blue refers to human cells (*n* = 4182), red refers to mouse cells (*n* = 2220), and gray refers to collisions (*n* = 45). (**E**) Proportion of HEK293T (blue) cells, NIH 3T3 (red) cells, or collisions (gray) detected by XYZeq for each column of the microwell array.

To overcome these limitations, we have developed XYZeq, a method that uses two rounds of split-pool indexing to encode the spatial location of each cell from a tissue sample into combinatorially indexed scRNA-seq libraries (*17*, *18*). Critical for the performance of XYZeq, we fixed tissue slices with dithio-bis(succinimidyl propionate) (DSP), a reversible cross-linking fixative that has been shown to preserve histological tissue morphology while maintaining RNA integrity for single-cell transcriptomics (*19*). In the first round of indexing, a fixed and cryo-preserved tissue section is placed on and sealed into an array of microwells spaced 500 μm center to center. The microwells contain distinctly barcoded reverse transcription (RT) primers (spatial barcode). This step physically partitions intact cells from tissue into distinct in situ barcoding reactions. After RT, intact cells are removed from the array, pooled, and distributed into wells for a second round of polymerase chain reaction (PCR) indexing, imparting each single cell with a combinatorial barcode (Fig. 1, A and B). After sequencing and demultiplexing, the spatial barcode maps each cell back to its physical location in the array (Fig. 1B). This combinatorial barcoding strategy theoretically could enable spatial transcriptomic analysis of large sets of single cells—with two rounds of split-pool indexing, 768 spatial RT barcodes, and 384 PCR barcodes, up to 294,912 unique single-cell barcodes can be generated.

To determine whether XYZeq can assign transcriptomes to single cells, we performed a mixed-species experiment where a total of 11 distinct ratios of DSP-fixed human [human embryonic kidney (HEK) 293T)] and mouse (NIH 3T3) cell mixtures were deposited into each of the 768 barcoded microwells, creating a cell proportion gradient along the columns of the array (Fig. 1C and Materials and Methods). XYZeq was used to generate scRNA-seq data for 6447 cells. A total of 94.8% of cell barcodes were assigned to a single species with an estimated barcode collision rate of 5.1% based on the percentage of cell barcodes with reads mapping to both human and mouse transcriptomes (fig. S1A). We hypothesized that a portion of collisions were due to contamination from ambient RNA released by damaged cells. Using DecontX (*20*), a hierarchical Bayesian method that assumes the observed transcript counts of a cell is a mixture of counts from two binomial distributions, we removed contaminating transcripts, reducing the collision rate to 0.7% (Fig. 1D and Materials and Methods). After computational decontamination and removal of collision events, we obtained a median of 939 unique molecular identifiers (UMIs) and 439 genes per human cell and 816 UMIs and 336 genes per mouse cell. Mapping each single cell to its originating microwell, we observed a high concordance between the observed and expected cell type proportions along the columns of the wells (Lin’s concordance correlation coefficient = 0.91; Fig. 1E and fig. S1B). Together, these results demonstrate that a minimal amount of barcode contamination takes place from single cells in each well and between neighboring wells on the array after pooling, indicating that the XYZeq workflow successfully produces spatially resolved scRNA-seq libraries.

**Fig. 2:**
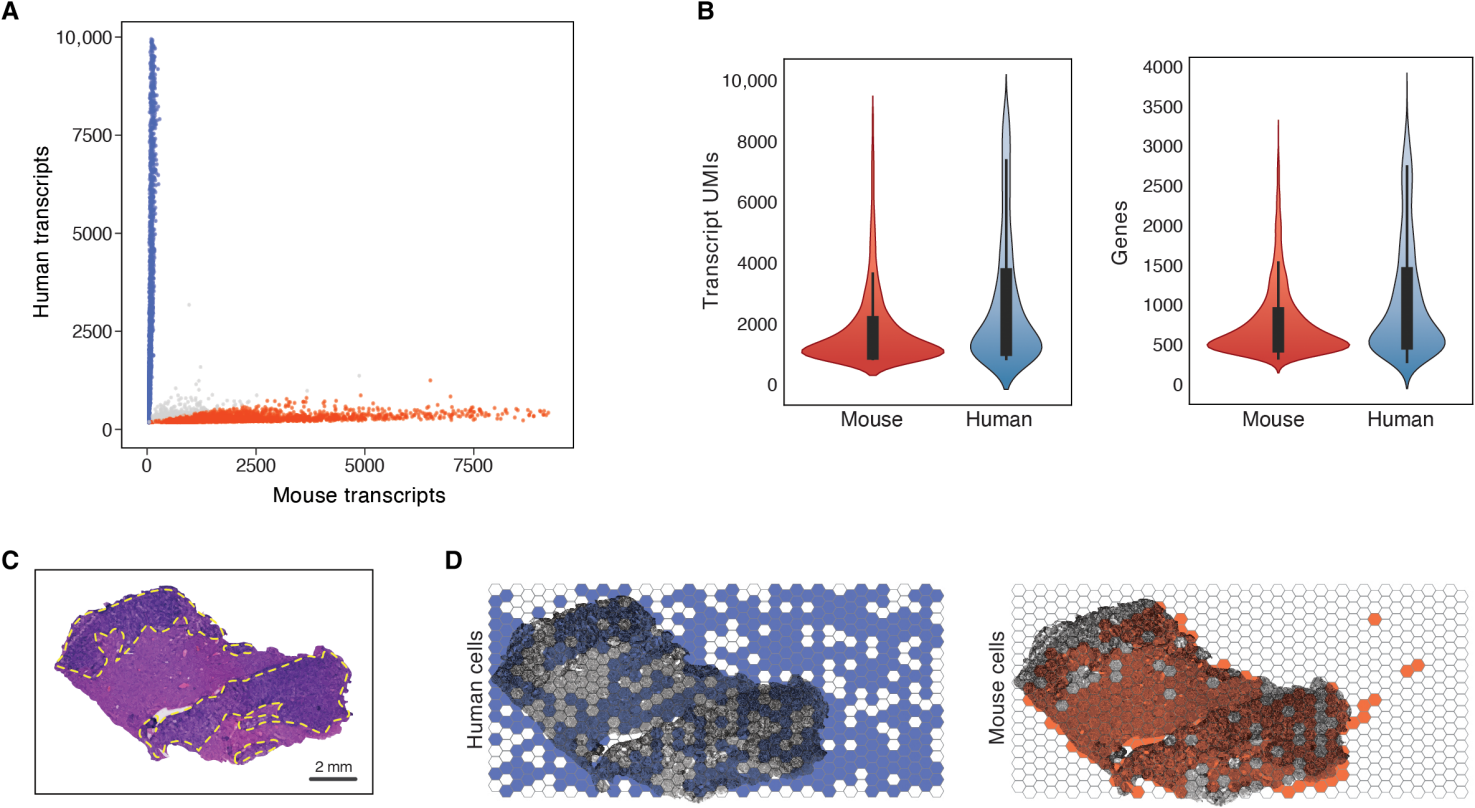
Spatially resolved single-cell transcriptomes captured from tissue. (**A**) Scatterplot of mouse (*x* axis) and human (*y* axis) UMI counts detected from liver/tumor tissues (*n* = 4) at 500 UMI cutoff after decontamination processing. Blue refers to human cells (*n* = 2657), red refers to mouse cells (*n* = 5707), and gray refers to collisions (*n* = 382). (**B**) Violin plots showing the number of detected UMIs (left) and genes (right) per mouse (red) and human (blue) cell. Median UMI counts for human cells: 1596; mouse cells: 1009. Median gene counts for human cells: 629; mouse cells: 456 across all liver/tumor slices. (**C**) H&E-stained image of the liver/tumor tissue slice. Tumor region, dark purple with yellow dotted outlines; liver region, pink. Scale bar, 2 mm. (**D**) Visualization of human (blue) and mouse (red) cell distribution on the XYZeq array overlayed on the H&E-stained slice.

We next applied XYZeq to a fixed and cryopreserved heterotopic murine tumor model established by intrahepatic injections of a syngeneic colon adenocarcinoma cell line, MC38, into immunocompetent mice. This model mimics tissue-infiltrating features of metastatic cancer and is associated with a relatively well-defined tumor boundary (*21*, *22*). MC38 tumor cells also have immunomodulating properties with previous data showing immune cells infiltrating the tumor/tissue interface approximately 10 days after tumor inoculation (*23*, *24*). Thus, we predicted that XYZeq could simultaneously capture the gene expression states and spatial organization of parenchymal liver cells, cancer cells, and tumor-associated immune cell populations. A 25-μm slice of fixed-frozen liver/tumor tissue from a C57BL/6 mouse was placed on top of the prefrozen microwell array while a sequential 10-μm slice was fixed for immunohistochemical staining (fig. S2A and Materials and Methods). We also deposited fixed human HEK293T cells into the same array at an average of 58 cells per well to serve as a mixed-species internal control to experimentally quantify collision rates. We performed XYZeq and observed an initial collision rate of 7.3% based on comparing the ratio of human versus mouse transcripts (fig. S2B). After computational decontamination and further quality control, which includes filtering cells based on cell counts and mitochondrial expression, the collision rate was reduced to 4.4% (Fig. 2A and Materials and Methods). After removing collisions, we obtained a total of 8746 cells and detected a median of 1596 UMIs and 629 unique genes per HEK293T cell and 1009 UMIs and 456 unique genes per cell from the heterotopic murine tumor model at 46% sequencing saturation (Fig. 2B). A hematoxylin and eosin (H&E)–stained serial section of the tissue revealed a histological boundary between the tumor and adjacent liver/tumor tissue (Fig. 2C). As expected, we observed HEK293T human cells distributed across the entire array, while mouse cells were sequestered within the boundary of the murine tissue (Fig. 2D). Note that empty spatial wells with no cells detected were likely due to a limited number of cells targeted for sequencing (~10,000). We obtained a median of 3 human cells per well and 9 mouse cells per well with a total of 13 cells per well expected (fig. S2C).

**Fig. 3:**
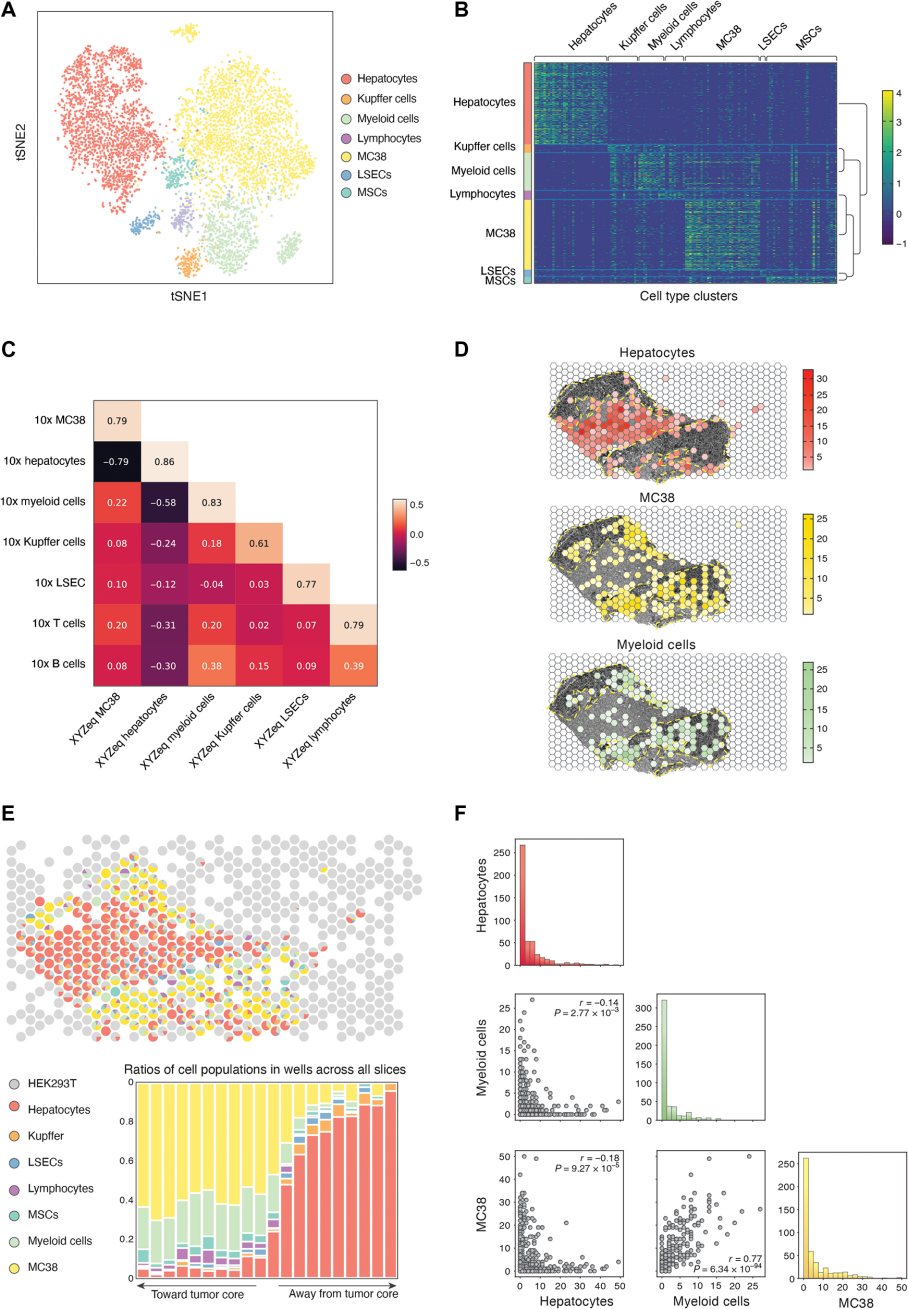
Frequency and spatial mapping of single-cell clusters from tissue. (**A**) tSNE visualization of the cell types identified from liver/tumor tissue. A total of 6623 total cells were plotted. (**B**) Heatmap of scaled marker gene expression and hierarchical clustering of genes that define each cell type from liver/tumor tissue. Reference for color bar in (A). (**C**) Correlations of pseudobulk expression values for matching cell types between XYZeq and 10x Genomics Chromium. (**D**) Spatial localization of hepatocytes, MC38, and myeloid cells overlaid on a bright-field image of tissue. Yellow dotted outline indicates tumor regions. (**E**) Pie chart of cell type composition for each XYZeq well from a representative liver/tumor tissue slice (top) and bar chart illustrating combined cell type composition across all four slices of liver/tumor tissue, which tracks with proximity to the tumor (bottom) (see Methods for proximity score). (**F**) Pairplot showing the frequency of hepatocytes, MC38, and myeloid cells in each well. Scatterplots show the colocalization of two cell types in each well. Histograms show the distribution of number of cells (*x* axis) per well (*y* axis) for each cell type. Pearson correlation (*r*) and *P* values are annotated.

XYZeq revealed distinct cell types within the murine liver and tumor. Semisupervised Leiden clustering revealed 13 cell populations in the murine tumor model (fig. S3A), from which seven cell types were annotated on the basis of markers that define each population: hepatocytes, cancer cells (MC38), Kupffer cells, liver sinusoidal endothelial cells (LSECs), mesenchymal stem cells (MSCs), lymphocytes, and myeloid cells (Fig. 3A). The annotation of MC38 tumor cells was supported by a high correlation of chromosomal copy numbers estimated from XYZeq scRNA-seq data and publicly available MC38 cytogenetic data (Pearson *r* = 0.78) (*25*). Notably, a partial amplification of chromosome 15 and a partial deletion of chromosome 14 observed in the XYZeq data were consistent with common chromosomal abnormalities seen in MC38 cells (fig. S3B). As a negative control, we saw low chromosomal copy number correlation when comparing MC38 cells to hepatocytes (*26*) and immune cells (*21*) (Pearson *r* = 0.05 and *r* = 0.17, respectively) (fig. S3B). A heatmap showing differentially expressed genes across seven cell types uncovered distinct clusters of cells defined by expression of canonical genes that are relatively exclusive to each cell type (Fig. 3B). Note that we estimated uniformly low rates of contamination of each cell cluster (median under 1%) with the exception of hepatocytes, which had a slightly higher rate at 2.2% (fig. S3C and Materials and Methods). We found comparable median UMIs and genes detected across all cell clusters including immune cell populations that have been difficult to profile using other combinatorial indexing methods (fig. S3, D and E) (*27*). Cell types expected in non–tumor-bearing liver were identified using markers previously described, which included hepatocytes, Kupffer cells, and LSECs (*26*). Consistent with the known heterogeneity of hepatocytes, we identified hepatocyte subsets annotated by the expression of pericentral markers (*Glul*, *Oat*, and *Gulo*) (fig. S3F) (*26*). MC38 adenocarcinoma cells comprised a large uniform cluster and were distinguished by the expression of the known marker *Plec* (*22*). Myeloid cells were defined by canonical markers *Cd11b* and *Cd74* (*28*), but other noncanonical markers were also observed, including *Myo1f* (*29*) and *Tgfb* (*30*). Lymphocytes showed a similar mix of broad and specific expression patterns of cell type markers, with expression of pan-lymphocyte marker *Il18r1*, T lymphocyte marker *Prkcq*, and cytotoxic T cell marker *Cd8b* (*31*–*33*). Last, we detected a cluster of MSCs/stromal cells that expressed both broad mesenchymal cell markers *Rbms3* and *Tshz2* and stem/stromal cell markers *Prkg1* and *Gpc6* (fig. S3F) (*34*–*38*).

We next assessed the reproducibility of XYZeq while comparing changes in the transcriptional landscape across the z-layer of the organ. Four nonsequential 25-μm tissue slices from the same frozen liver/tumor sample block were processed and analyzed. The average expression over all cells for genes detected across all slices was highly correlated between each pair of slices (average pairwise Spearman *r* = 0.93) (fig. S4A). We noted that among the four tissue sections, slices 1 and 2, which were the two most proximal slices in their *z* coordinates (separated by 80 μm), had the highest expression correlation (Spearman *r* = 0.96). In contrast, slices 1 and 4, which were the most distal in *z* coordinates (separated by 830 μm), had the lowest correlations (Spearman *r* = 0.91). Further, clusters jointly annotated across all four slices consisted of cells from each slice, suggesting that the observed heterogeneity is not due to batch effects (fig. S4B).

We further compared the quality of the scRNA-seq data generated by XYZeq to another single-cell technology that is commercially available. To accomplish this, we compared the cell type clusters identified from XYZeq to those identified from an independent scRNA-seq dataset of the same liver/tumor model generated using the 10x Genomics droplet-based Chromium system. Most cell populations detected by 10x were also observed by XYZeq, except neutrophils, erythroid progenitors, and plasma cells (Fig. 3C and fig. S5A), which are immune cell populations known to be sensitive to the cryopreservation (*39*) required for XYZeq. 10x did not capture MSCs even though cells were isolated from fresh liver/tumor samples. In addition, B cells identified using the 10x platform correlated with the myeloid population detected by XYZeq, likely due to the transcript capture of *Ly86*, *Cd74*, and several class II histocompatibility antigen genes (e.g., *H2ab1* or *H2dmb1*). For the six cell types identified in both the 10x and XYZeq data, we observed high correlations in both the cell type proportions (Lin’s concordance correlation coefficient = 0.99; fig. S5B) and the pseudobulk expression profiles of each cell type (Pearson *r* = 0.64 to 0.86, *P* < 0.01; Fig. 3C).

Next, we turned to the critical question of whether XYZeq can determine the spatial location of each cell. To do this, we compared the spatial localization of each cell cluster to the images of H&E-stained sequential slices. First, to determine that we could accurately define liver from tumor tissue, we confirmed that the density of hepatocytes and cancer cells across the spatial wells overlap with the histological annotation of the adjacent section (Fig. 3D). Projection of other cell types revealed distinct spatial organization patterns for myeloid cells, lymphocytes, Kupffer cells, MSCs, and LSECs (Fig. 3D and fig. S6A). Quantification of cellular composition occupying each spatial well revealed that MSCs, lymphocytes, and myeloid cells were colocalized with cancer cells, while Kupffer cells and LSECs colocalized with hepatocytes, suggesting potential regions of cellular interaction in tumor-infiltrated tissue (Fig. 3E and Materials and Methods). These qualitative observations were confirmed by pairwise correlation analysis of cell type proportion across all the wells (0.37 ≤ Pearson *r*≤ 0.77, *P* < 0.05; Fig. 3F and fig. S6B).

To assess the generalizability of XYZeq to other tissues, we processed samples from the same heterotopic murine tumor model in the spleen. We recovered a total of 7505 cells at a median of 1312 UMIs and 661 unique genes per HEK293T cell and 1169 UMIs and 577 unique genes per mouse cell at an estimated collision rate of 1.36% (fig. S7, A and B). Similar to the liver/tumor model, XYZeq was able to reconstruct the boundaries of the splenic mouse tissue with the MC38 tumor region annotated on a sequential H&E-stained slice (fig. S7, C to E). A median of four human cells per well and seven mouse cells per well were detected (fig. S7F). Semisupervised Leiden clustering revealed six distinct cell populations for the spleen/tumor model including B cells, T cells, myeloid cells, MSCs, endothelial cells, and MC38 tumor cells (fig. S8A). We observed that all four spleen/tumor slices contributed to each cell type cluster, suggesting that the annotated clusters are not due to batch effects (fig. S8B). A heatmap showing differentially expressed genes across the six cell types revealed distinct clusters of cells expressing canonical genes that are relatively exclusive to each type (fig. S8C). Cells from each type could be spatially mapped across the tissue (fig. S8D). Collectively, these results demonstrate that XYZeq can generate spatially resolved scRNA-seq data from different fixed-frozen tissues.

**Fig. 4:**
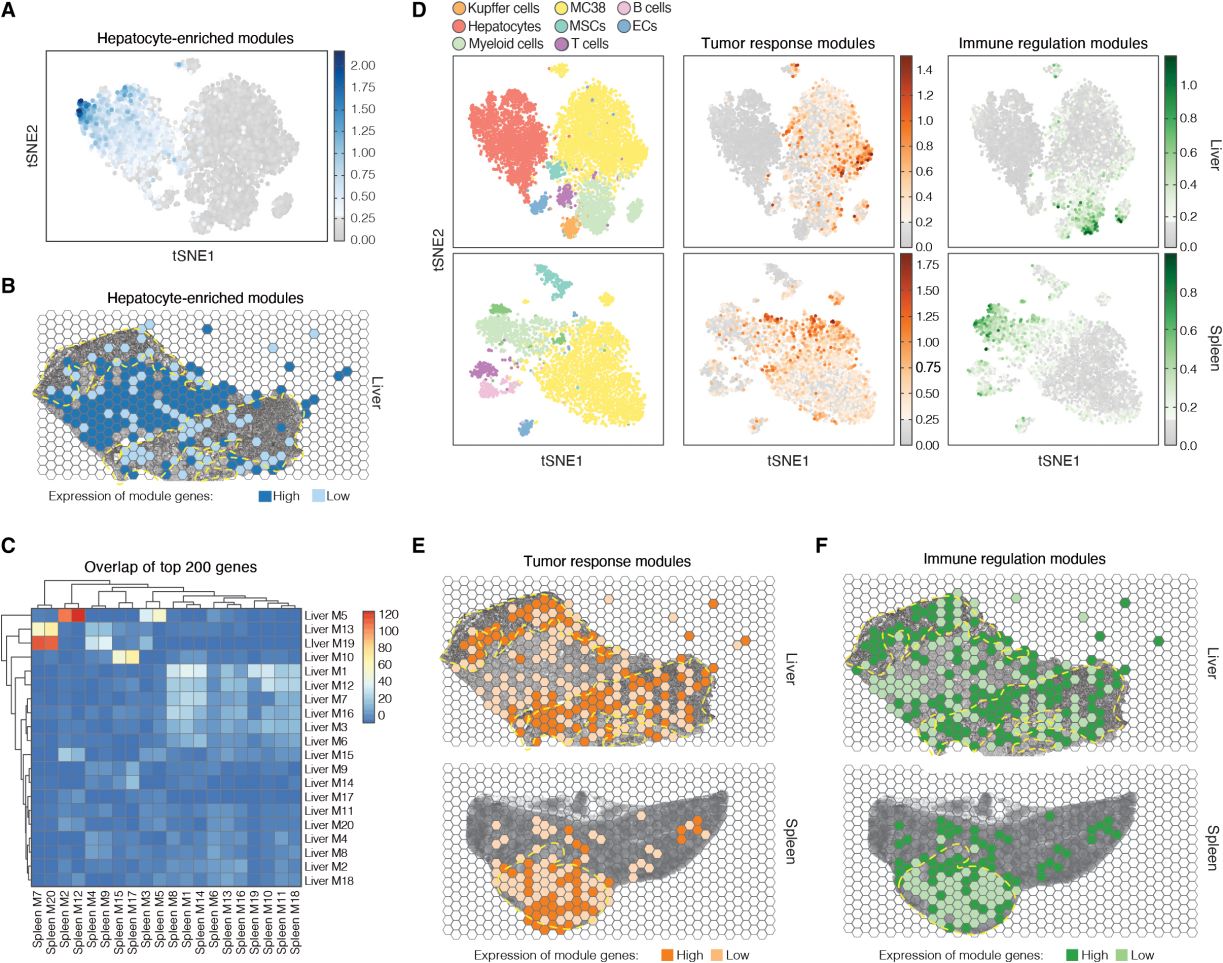
Expression of gene modules in space that track with cellular composition. (**A**) Projection of average expression of hepatocyte-enriched module (LM14) in tSNE space. Each dot is a cell and colored by the average expression of top contributing module genes (Materials and Methods). (**B**) Spatial expression of hepatocyte-enriched module (LM14). Each spatial well is colored by the average expression of the top contributing module genes weighted by the number of cells per well. Wells are binarized into high (above weighted average) versus low (all other nonzero expression). Yellow dotted outlines indicate tumor regions. (**C**) Heatmap representing the number of overlapping genes between each pair of modules in liver/tumor and spleen/tumor. Each row is an LM, and each column is an SM. (**D**) tSNE projection of XYZeq scRNA-seq data colored by annotated cell types in liver/tumor (top left) and spleen/tumor (bottom left) and mean gene expression of the top overlapping modules between liver/tumor (top row) and spleen/tumor (bottom row). Tumor response modules correspond to LM5 and SM12, and immune regulation modules correspond to LM19 and SM7. ECs, endothelial cells. Spatial projection visualizes the mean expression of the tumor response modules (**E**) corresponding to LM5 and SM12 and the immune regulation modules (**F**) corresponding to LM19 and SM7. Each well in (E) and (F) is colored by the average gene expression of each module weighted by the number of cells per well (high versus low), and yellow dotted outline indicates tumor regions. Wells are binarized into high (above weighted average) versus low (all other nonzero expression).

The ability to obtain spatial and single-cell transcriptomic data simultaneously allowed us to assess the effects of cellular composition on gene expression patterns across space. We applied non-negative matrix factorization (NMF) to both the liver/tumor and spleen/tumor scRNA-seq data to define modules of coexpressed genes and associated the expression of each module in each cell type with its expression across spatial wells. Using our approach, we identified 20 modules of coexpressed genes in each tissue (Materials and Methods). As a proof of principle of the approach, we first identified liver module (LM) 14 from the liver/tumor data, which was predominantly expressed by the hepatocyte cluster in the t-distributed stochastic neighbor embedding (tSNE) space (Fig. 4A). As expected, the highest LM14-expressing wells were enriched for hepatocytes, suggesting that the spatial variability of this module is largely driven by the frequency of hepatocytes (Fig. 4B).

Next, we reasoned that because both the liver and spleen were injected with the same tumor cell line, the invading tumors may induce a shared gene expression profile that vary over space, driven in part by the cellular composition of the tumor microenvironment. To test this hypothesis, we first identified pairs of matching gene modules between the two tissues from the NMF analysis (Materials and Methods). We found four distinct LMs that had at least 25% of genes overlapping with spleen/tumor modules (SMs) (Fig. 4C and fig. S9A). Gene ontology analysis of the modules revealed the enrichment of genes implicated in tumor response, immune regulation, and cell migration (figs. S9, B and C, and S10B). Consistent with the enrichment analysis, many of the genes from these modules have been implicated in tumorigenesis (complete gene lists are in table S1). Unlike LM14, further analysis of these matching modules revealed a heterogeneous composition of cell populations that contributed to the expression of specific module genes (fig. S9D and Materials and Methods). For example, the tumor response module LM5 and its matching modules SM2 and SM12 (Fig. 4C and fig. S9A) consisted of genes predominantly expressed in MC38 tumor cells with some expression in myeloid cells and lymphocytes (Fig. 4D, fig. S9D, and Materials and Methods). The immune regulation modules, LM13 and LM19 (matched with SM7 and SM20), consisted of genes expressed primarily in both conventional (e.g., myeloid and lymphocytes) and nonconventional (e.g., Kupffer cells from liver samples) immune cells (Fig. 4, C and D, and fig. S9D). The expression of these overlapping modules was highest in regions densely infiltrated with cancer cells (Fig. 4, E and F). Collectively, these results show that the joint analysis of scRNA-seq and spatial metadata from XYZeq can identify spatially variable gene modules due to differences in cellular composition across tissue samples.

**Fig. 5:**
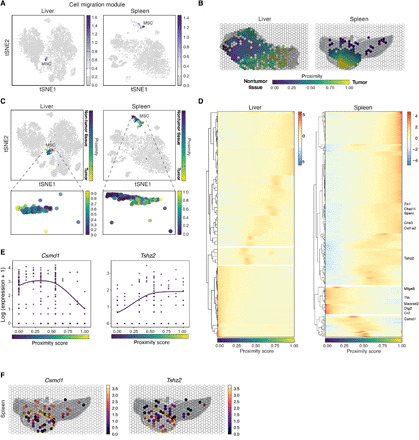
Differential gene expression within MSCs associated with their spatial proximity to tumor. (**A**) Average expression of the cell migration modules (LM10 and SM17) in tSNE space. Each dot is a cell colored by its mean expression of the top module genes between corresponding liver/tumor and spleen/tumor modules. (**B**) XYZeq array colored by the tumor proximity score. Values near 1 (yellow) indicate regions rich in tumor, values near 0 (purple) indicate regions rich in nontumor cells, and wells capturing the border between the two tissue types take on values around 0.5 (blue/green). (**C**) MSCs colored by the cell-specific proximity score in tSNE space. (**D**) Row-clustered heatmap showing the scaled, mean gene expression in MSCs of genes enriched in three spatial regions (intratumor, boundary, and intratissue) along the one-dimensional proximity score. For spleen/tumor, statistically significant genes enriched in the tumor and nontumor regions are highlighted. (**E**) Log expression (*y* axis) of *Csmd1* (left) and *Tshz2* (right) along the proximity score (*x* axis). Each dot corresponds to one MSC cell, and the regression line is fitted using the negative binomial distribution (Materials and Methods). (**F**) Projection in space of mean expression of *Csmd1* (left) and *Tshz2* (right) in MSCs. Yellow dotted outline indicates tumor region.

We next focused our analysis on matching modules LM10 and SM15/SM17, which are primarily expressed by MSCs and enriched for genes involved in cell migration (Figs. 4C and 5A and figs. S9D and S10, A and B). Because MSCs are known to have homing abilities to injured or inflamed sites (*40*), we hypothesized that LM10 could be differentially expressed in MSCs based on their proximity to the tumor. To test this hypothesis, we first computed a tumor proximity score for each well based on the composition of and distance from nearby wells (Fig. 5B; see Materials and Methods and fig. S11 for score definition). Projecting the proximity score onto MSCs in tSNE space revealed that the transcriptional heterogeneity of the population is associated with spatial proximity to tumor (Fig. 5C). We then analyzed the MSC expression profiles using tradeSeq (*41*) to identify differentially expressed genes that tracked with the proximity score. We identified and clustered 177 genes from the liver/tumor tissue (*P* < 0.05) and 66 genes from the spleen/tumor tissue (*P* < 0.05) that are associated with the continuous, one-dimensional proximity score (Fig. 5D). The genes were broadly divided into three groups based on the proximity cells to tumor: intratumor, tumor-tissue boundary, and intratissue with statistically significant genes highlighted for the spleen/tumor tissue (Benjamini-Hochberg false discovery rate < 0.05) (Fig. 5D). For MSCs found in the intratumor regions of the spleen/tumor, many of the differentially expressed genes are reported to regulate the extracellular matrix (ECM) (Fig. 5D, right) (*42*–*45*), suggesting that MC38 cells may induce a local gene expression program in neighboring MSCs that could contribute to malignant remodeling of the ECM.

Last, we leveraged the scRNA-seq data from XYZeq to visualize how individual MSCs expressed *Tshz2* and *Csmd1*, two genes of divergent function that are spatially variable with respect to the tumor in the spleen. Both genes are characterized as tumor suppressor genes and are often silenced in cancer cells to promote malignant growth and metastasis (*36*, *46*, *47*). However, we found that spleen/tumor MSCs expressed lower levels of *Csmd1* but higher levels of *Tshz2* in closer proximity to the tumor (Fig. 5E). The mean differential expression of these genes was specific to splenic MSCs and not expressed by MC38 tumor cells. The expression pattern of each of these genes in space revealed a pattern consistent with the aforementioned spatial trajectory analysis, suggesting that their heterogeneous expression in MSCs may be determined by the location of the cells with respect to tumor (Fig. 5F). Together, these results reveal that joint analysis of spatial and single-cell transcriptomic data from XYZeq can detect transcriptionally variable genes within specific cell types (e.g., MSCs) driven by their location within the complex tissue architecture.

## DISCUSSION

We introduce XYZeq, a new scRNA-seq workflow that encodes spatial meta information at 500-μm resolution. XYZeq enables unbiased single-cell transcriptomic analysis to capture the full spectrum of cell types and states while simultaneously placing each cell within the spatial context of complex tissue. In murine tumor models, we demonstrate that XYZeq identifies both spatially variable patterns of gene expression determined by cellular composition and heterogeneity within a cell type determined by spatial proximity. Looking forward, XYZeq provides a scalable workflow that can be adapted to multiple z-layers of tissue and can potentially facilitate analysis of entire organs. Large-scale integrated profiling of multiple modalities of single cells mapped to the structural features of their tissue will enable greater understanding of how the tissue microenvironment affects cellular infiltration and interaction in health and disease.

## MATERIALS AND METHODS

### Mice, tumor cell line, and tumor inoculation

Six- to 12-week-old C57BL/6 female mice were purchased from Jackson Laboratories and housed under specific pathogen–free conditions. MC38 colon adenocarcinoma cell line expressing luciferase was a gift from R. D. Beauchamp (Vanderbilt University). MC38 cell line was cultured in complete cell culture medium (RPMI 1640 with GlutaMAX, penicillin, streptomycin, sodium pyruvate, Hepes, non-essential amino acid, and 10% fetal bovine serum). Cell lines were routinely tested for mycoplasma contamination. For experiments, mice were given an anesthetic cocktail of buprenorphine (300 μl) and meloxicam (300 μl) 30 min before the procedure. At the time of surgery, one drop of bupivacaine was administered, and mice were anesthetized with isoflurane before intrahepatic (or intrasplenic) injection of MC38 colon adenocarcinoma cells (50 μl at 10 × 10^6^ cells/ml) using a 30-gauge ^1^/_2_-inch needle. Incision was stapled closed, and postoperative care was given to the mice. All experiments were conducted in accordance with the animal protocol approved by the University of California, San Francisco Institutional Animal Care and Use Committee.

### Cancer model system

The intrahepatic and intrasplenic cancer model that we used for the paper is described in great detail in a recently published report by Lee *et al.* (*21*). Briefly, intrahepatic and intrasplenic tumors were generated by subcapsular injection of the tumor cells directly into the organs. To establish the ideal time point for sacrificing the mice, in vivo imaging was done on tumor-inoculated mice. Intraorgan-injected MC38 cells were modified to express the firefly luciferase. Mice were intraperitoneally infected with d-luciferin (150 mg/kg; Gold Biotechnology) 7 min before imaging with the Xenogen IVIS Imaging System. Mice with detectable tumor nodules with at least 5-mm fluorescence were euthanized for tissue harvesting. Organs to be used for XYZeq were fixed with DSP (Thermo Fisher Scientific) and cryopreserved, while organs used for 10x Genomics Chromium single-cell sequencing were digested in RPMI 1640 complete medium that were supplemented with collagenase D (125 U/ml; Roche) and deoxyribonuclease I (20 mg/ml; Roche) and then processed for single-cell suspension using the gentleMACS tissue dissociator per the manufacturer’s protocol (Miltenyi).

### 10x Genomics Chromium platform

Cells isolated from tissue were washed and resuspended in phosphate-buffered saline with 0.04% bovine serum albumin at 1000 cells/μl and loaded on the 10x Genomics Chromium platform per the manufacturer’s instructions and sequenced on NovaSeq or HiSeq 4000 (Illumina).

### Tissue harvesting and cryopreservation

At day 10 after tumor inoculation, mice were euthanized and harvested for the tumor-injected liver (or spleen) and incubated for 30 min in ice-cold dimethyl sulfoxide–free freezing media (Bulldog Bio). This was followed by 30 min of incubation in ice-cold DSP (Thermo Fisher Scientific) supplemented with 10% fetal calf serum (FCS) and then neutralized in ice-cold 20 mM tris-HCl (pH 7.5). The organs were placed in a cryomold, sealed airtight, and slowly frozen overnight at −80°C.

### Cells and reagent dispensing into array

The sciFLEXARRAYER S3 (Scienion AG) was used to dispense cells and reagents to the microwell arrays. Drop stability and array quality were assessed for each experiment. Before dispensing into the microwell arrays slides, Autodrop detection was used to assess drop stability and quantify the velocity, deviations, and drop volume for each reagent. Volume entry was used to determine the number of drops required to reach the total designated well volume. Each well oligo(dT) primer (5′-CTACACGACGCTCTTCCGATCTNNNNNNNNNN[16–base pair unique spatial barcode] TTTTTTTTTTTTTTTTTT-3′, where “N” is any base; IDT) was spotted into a different well in the array. During barcoding, the dew point control software monitored the ambient temperature and humidity, allowing dynamic control of the temperature of the source plate to maintain nominal oligo concentrations through the duration of the run. Barcoded slides were dried in the wells before storage. Reaction mix (Thermo Fisher Scientific) was added to wells and automated with a 10% bleach wash between each probe to eliminate carryover contamination. Dissociation/permeabilization buffer was printed into each well on the day of experiment, and tissue section was loaded onto the microwell array slides. For all tissue experiments, DSP-fixed HEK293T cells were added at 5 μl (at 10 × 10^6^ cells/ml) to the RT digestion mix before being dispensed across all the wells in the microarray. The average number of HEK293T cells were 58 cells per well; however, the absolute number of cells per well likely varied across the array due to the cells being in suspension inside the dispensing nozzle. Cells harvested from the array after incubation were analyzed on the Aria (BD Biosciences), and datasets were analyzed using FlowJo software (Tree Star Inc.).

### Array fabrication

Photoresist masters are created by spinning on a layer of photoresist SU-8 2150 (Thermo Fisher Scientific) onto a 3-inch silicon wafer (University Wafer) at 1500 rpm and then soft baking at 95°C for 2 hours. Then, photoresist-layered silicon wafer is exposed to ultraviolet (UV) light for 30 min over a photolithography mask (CAD/Art Sciences, USA) that was printed at 12,000 DPI (dots per inch). After UV exposure, the wafers are hard-baked at 95°C for 20 min and then developed for 2 hours in fresh solution of propylene glycol monomethyl ether acetate (Sigma-Aldrich) to develop, followed by a manual rinse with fresh propylene glycol monomethyl ether acetate then baked at 95°C for 2 min to remove residual solvent. Polydimethylsiloxane (PDMS) mixture (Sylgard 184, Dow Corning, Midland) with pre-polymer:curing agent ratio of 10:1 was poured over the SU-8 silicon wafer master. This was placed in a 100-mm petri dish and was cured overnight in a 70°C oven. This PDMS-negative mold was peeled off the SU-8 silicon master the following day. PDMS block was placed on a flat surface, and Norland Optical Adhesive 81 (NOA81) (Thorlabs) was poured into the mold to cover the entire surface. A slide was placed on top of the NOA-poured PDMS mold, and a transparent weight was placed on top. NOA was cured for 2 min under UV light, flipping once halfway through the UV curing time. Last, the PDMS mold was detached from the cured NOA microwell array slide (referred to as microwell array chips). The dimensions of each hexagonal well are approximately 400 μm in height and 500 μm in diameter with the volume of 0.04 mm^3^, which can hold 40 nl of liquid.

### XYZeq methodology

Liver/tumor organ was mounted on a cyrostat (Leica) and sliced at 25 μm for use as an XYZeq experimental sample or mounted on a histology slide at 10 μm for immunohistochemical staining. On the day of experiment, XYZeq microwell array chips were spotted with an RT cocktail mix that was spiked-in with DSP-fixed HEK293T cells. The microwell array chips were brought down to −80°C, and a tissue slice was placed on top of the array. A digital image was taken to document the orientation of the tissue before sandwiching a silicone gasket sheet between the XYZeq microwell array chip and a blank histology slide. The chip was placed in a microarray hybridization chamber (Agilent) to ensure an airtight seal while undergoing tissue digestion and RT. To recover high-quality RNA from fixed-frozen tissue, the microarray hybridization chamber housing the chip had to undergo a gradual step-wise temperature increase to 42°C before the 20-min incubation to undergo RT. The chip was removed from the chamber and placed in a 50-ml conical tube with 50 ml of 1× SSC buffer and 25% FCS. The tube was vortexed and spun down at 1000 rcf for 10 min. Excess volume was removed, and cells were filtered and stained for DAPI (4′,6-diamidino-2-phenylindole; Life Technologies) before sorting (BD Aria) into 96-well plates preloaded with 5 μl of the second RT mix. Plates were reverse-transcribed for 1.5 hours at 42°C, followed by PCR using 2× Kapa HotStart ReadyMix (Kapa Biosystems). PCR amplification was performed with an indexing primer (5′-AATGATACGGCGACCACCGAGATCTACAC [i5]ACACTCTTTCCCT ACACGACGCTCTTCCGATCT-3′; IDT). Contents of the PCR plate were pooled into 2-ml Eppendorf tubes, and complementary DNA (cDNA) was purified with AMpure XP SPRI bead (Beckman). cDNA was tagmented and amplified with Illumina Nextera library p7 index (IDT). Final library was analyzed by BioAnalyzer (Agilent) and quantified by Qubit (Invitrogen) and sequenced on a NovaSeq or HiSeq 4000 (Illumina) (read 1: 26 cycles; read 2: 98 cycles; index 1: 8 cycles; index 2: 8 cycles).

### XYZeq decontamination analysis

In our analysis, we recognized that some reads aligning to the mouse genes were present in cells that otherwise had high alignment to the human genome. We suspected that these reads were ambient RNA contamination and sought to remove them. We first removed mouse-aligned transcripts with an extremely high expression in human cell population [*n* = 59, log(counts +1) > 6]. The human cell population was considered a control in the contamination detection, because any ambient RNA from lysed cells was expected to contaminate both mouse and human cells. DecontX (*20*) was then performed to estimate the contamination rate for different cell populations using the human-mouse mixture dataset and therefore derive a decontaminated count matrix from the raw data. Briefly, the algorithm applies variational inference to model the observed counts of each cell as a mixture of true gene expression of its corresponding cell population and the contamination signature (from other cell populations) and then subtracts the contamination signature (fig. S3C). By considering the human-mouse mixed-species experiment, we could remove those counts potentially contributing to collision and effectively account for all potential transcripts in the lysed cells that contribute to ambient RNA. In fig. S3C, the initial estimated contamination rate for each mouse cell type is plotted with the median estimates ranging from 0.06 to 0.31%, with the highest seen in the hepatocyte cell cluster with 2.18% initial contamination fraction. All the downstream analysis was performed on the basis of the decontaminated data after contamination removal.

### How distinctions were made between collision rate and contamination rate

The collision rate is directly calculated from the gene expression of human-mouse mixture dataset based on the ratio between mouse-aligned and human-aligned transcripts, while the contamination rate for each cell is estimated as a cell-specific parameter in the Bayesian hierarchical model via variational inference from DecontX. To specify the contamination rate, each cell has a beta-distributed parameter modeling its proportion of transcript counts, which come from its native expression distribution. The estimated contamination rate for each cell is the proportion of transcript counts, which come from contamination in the Bayesian model. Each transcript in a cell follows a multinomial distribution parameterized by the native expression distribution of its cell population or contamination from all the other cell populations, given a Bernoulli hidden state, indicating whether the transcript comes from its native expression distribution or from the contamination distribution.

### Cell species mixing experiment

Mixture of HEK293T and NIH 3T3 cells were deposited into wells in a gradient pattern across the columns of the array with a total of 11 distinctive cell proportion ratios. Specifically, columns on the array were spotted with human cell–to–mouse cell ratio of 100/0; 90/10; 80/20; 70/30; 60/40; 50/50; 40/60; 30/70; 20/80; 10/90; 0/100; 10/90; 20/80; 30/70; 40/60; 50/50; 60/40; 70/30; 80/20; 90/10; and 100/0, with only human cells flanking the end columns and only mouse cells in the center columns. The ratio of UMI deduplicated reads aligning to either human or mouse reference genomes was calculated for each cell, and those with less than 66% aligning to a single species were deemed barcode collision cells.

### XYZeq single-cell analysis

Single-cell RNA sequence data processing was performed where sequencing reads were processed as previously described (*17*). Briefly, raw base calls were converted to FASTQ files and demultiplexed on the second combinatorial index using bcl2fastq v2.20. Reads were trimmed using trim galore v0.6.5, aligned to a mixed human (GRCh38) mouse (mm10) reference genome and UMI deduplicated. Reads were then assigned to single cells by demultiplexing on the first combinatorial index, before the construction of a gene by cell count matrix. The count matrix was processed using the Scanpy toolkit. Cells with less than 500 UMIs and greater than 10,000 UMIs, as well as cells expressing less than 100 unique genes or more than 15,000, were discarded. Cells with more than 1% mitochondrial read percentage were also discarded. Gene counts were normalized to 10,000 per cell, log-transformed, and further filtered for high mean expression and high dispersion using the filter genes dispersion function, with a minimum mean of 0.35, maximum mean of 7, and minimum dispersion of 1. Gene counts were then corrected using the regress out function with total counts per cell and the percentage mitochondrial UMIs per cell as covariates. Subsequent dimensionality reduction was done by scaling the gene counts to a mean of 0 and unit variance, followed by principal components analysis, computing of a neighborhood graph, and tSNE. Leiden clustering was performed with a resolution of 0.8, and cells were grouped to reveal distinct murine cell types and human HEK293T cells.

### 10x data processing

Count matrices were generated using the “count” tool from Cell Ranger version 3.1.0, using the combined human and mouse reference dataset (version 3.1.0) and the “chemistry” flag set to “fiveprime.” The count matrix was processed using the Scanpy toolkit. Cells with less than 500 UMIs and greater than 75,000 UMIs, as well as cell expressing less than 100 unique genes and greater than 10,000, were discarded. Cells with more than 7.5% mitochondrial read percentage were also discarded. Gene counts were normalized to 10,000 per cell, log-transformed, and further filtered for high mean expression and high dispersion using the filter genes dispersion function, with a minimum mean of 0.2, maximum mean of 7, and minimum dispersion of 1. Gene counts were then corrected using the regress out function with total counts per cell and the percentage mitochondrial UMIs per cell as covariates. Subsequent dimensionality reduction was done by scaling the gene counts to a mean of 0 and unit variance, followed by principal components analysis, computing of a neighborhood graph, and tSNE. Leiden clustering was performed with a resolution of 1, and cells were grouped to reveal major murine cell types and human HEK293T cells.

### Heatmap for XYZeq

Mouse cells were subsetted from the XYZeq processed data matrix. The processed gene expression values were plotted in a heatmap with a minimum fold change of 1.5 and hierarchically clustered using the heatmap function from Scanpy, with the default settings of Pearson correlation method and complete linkage.

### XYZeq gene pairplot

Four slices of liver/tumor tissue were processed using the XYZeq assay (with HEK293T cells spiked in) and aligned to a joint human and mouse reference. All genes with at least one count in each slice were kept, and the counts across the common set of genes between pairwise slices were plotted in the lower triangle, with the Spearman correlation for the data shown in the upper triangle. Along the diagonal, histograms were plotted showing the distribution of counts per gene for all the nonzero genes for each slice.

### XYZeq cells per well pairplot

Pairplot shows the number of microwells containing pairwise combinations of cell types. For scatterplots, each point in the plot represents a well, and its coordinate positions indicate the number of cells of each cell type present in that well. Every dot on the scatterplot is a gene representing mean per gene for common genes across all cells in the slices. Along the diagonal of the figure are histograms, showing the univariate distribution of cell number per well for the given cell type.

### Heatmap comparing 10x to XYZeq

Mouse cells were subsetted from each of the processed data matrices. For pairwise mouse Leiden clusters found between XYZeq and 10x, the scaled and log-transformed gene expression values of common genes were plotted. For each comparison, a Pearson correlation was calculated and plotted in the heatmap. Row/column labels were ordered according to their corresponding cell types.

### Correlation plot

Mouse cells were subsetted from each of the processed data matrices. Proportions for each cell type (as determined by the Leiden clustering and visualized using tSNE) were plotted, and the coefficient of determination was calculated by fitting to the model that assumes proportions are equal between the two assays.

### Gene module analysis of top contributing genes

To identify gene modules using NMF, genes expressed in fewer than five cells and cells expressing fewer than 100 genes were filtered out. Variance stabilizing transformation was performed on count data, and confounding covariates including number of counts per cell, batch, and mitochondrial read percentage were regressed out by a regularized negative binomial regression model using the SCTransform (*48*) function in the Seurat R package. Pearson residual values from the regression model were centered, and all negative values were converted to zero. Nonsmooth NMF (nsNMF) was performed on the resulting expression data with a rank value of 20 using the nmf (*49*) function in NMF R package. In each module, genes were sorted by their magnitude in the corresponding coefficient matrix in a descending order. Gene ontology enrichment analysis was performed for the sorted genes in each module using GOrilla (*50*). For each module, the top consecutive genes with higher coefficients in this module compared to all the other modules were further selected as genes contributing the most to the module (*51*) in the tissue-specific analysis. Binary spatial plots were generated by first calculating the median expression across all the cells for each well within each batch based on the log-normalized gene expression data. We then extracted the mean expression across all the genes within one module for each well and calculated the average of mean expression across selected module genes for each well weighted by the number of cells in each well. The wells with a mean expression across genes above the weighted average were labeled as highly expressing for that gene module, and all the other wells with nonzero expression of those selected module genes were labeled as lowly expressing that gene module. tSNE plots representing the gene modules were colored by their mean expression of genes within the annotated module.

### Overlapping analysis between the gene modules identified in liver/tumor and spleen/tumor

Gene modules were first identified using nsNMF with a rank value of 20 for the two tissues, liver/tumor and spleen/tumor, respectively. The top 200 genes in each sorted gene list for a module were selected as having high association with the module. For each module in the liver/tumor tissue, the spleen/tumor module with the largest gene overlap was initially matched as functionally similar. We then removed those matched pairs with fewer than 25% overlapping genes out of top 200 genes in the liver/tumor module. To calculate cell type fractions that make up each module, the average gene expression for each gene across all the cells was calculated. Median expression across all the overlapping genes for each cell type was further computed, which was later transformed into fractions by dividing by the sum of median expression across all the cell types.

### Defining the proximity score by wells

We sought to define a score for each well of the hexagonal well array that would capture how centrally located a well was within either the tumor or nontumor tissue domains. Central to the method was the determination of successive concentric “layers” of wells that were adjacent to a well in question: those corresponding to its immediate neighbors (layer 1), those wells exactly two wells away (layer 2), and so on, for *n* layers. In the spleen/tumor, we selected several wells on the far side of the tumor region and set the score of these wells to 1. We then took 10 successive layers of wells and decreased the score linearly with each layer, with the wells in layers 10 and beyond set to 0. In the liver, MC38 cells were found in different locations, and therefore, unlike the spleen, there was no single unidirectional spatial dimension to place all MC38 cells at one end and all nontumor tissue cells at the other. Therefore, we used an alternative approach to calculate these scores in the liver/tumor tissue. For each well *w*_*x*, *y*_, annotated by their *x*, *y* position on the hexagonal well array, we calculated the proportion of hepatocytes, *p*_*x*,*y*_, since the hepatocytes were the most abundant parenchymal cell type in and strictly associated with the nontumor liver tissue

$$t_{x,y}=\#\text{of total hepatocytes and MC38 cells in}\;w_{x,y}$$

$$h_{x,y}=\#\text{of hepatocytes in}\;w_{x,y}$$

$$p_{x,y}=\frac{h_{x,y}}{t_{x,y}}$$

Then, for each well in question *w*_*x*,*y*_, we tabulated the surrounding wells in each of the successive concentric 10 layers. We denote these wells *w*_*x*^′^*y*^′^_ to differentiate from the well in question. For each of those layers *l*, we took its constituent wells’ *p*_*x*^′^,*y*^′^_ and calculated a cell number–weighted average *p*_*x*,*y*, *l*_

$$w_{x,y,l}=\{w_{x\prime y\prime }\in \text{layer}\;l\;\text{of}\;w_{x,y}\}$$

$$t_{x,y,l}=\#\text{of total hepatocytes and}\;\text{MC}38\;\text{cells in}\;w_{x,y,l}$$

$$p_{x,y,l}=\sum_{x\prime ,y\prime }^{w_{x,y,l}}\frac{t_{x\prime ,y\prime }}{t_{x,y,l}}p_{x\prime ,y\prime }$$

Then, for the well in question *w*_*x*,*y*_, we calculated a distance-weighted average of all the *p*_*x*,*y*,*l*_, and this became the proximity score *s*_*x*,*y*_ for the well in question. The distance weights for each layer, *u_l_*, were based on an exponential decay, terminated to 10 terms and then normalized to 1 by dividing by the sum of all weights *u*_s_. We give equal weight to *p*_*x*,*y*_ and the value for the layer 1 neighbors *p*_*x*,*y*,1_. A decay factor *d* of 1.05 was chosen empirically, as it seemed to create the most uniform-like distribution of the scores across all wells

$$d=1.05,u_{s}=\sum_{l=1}^{10}\frac{1}{d^{l}}$$

$$u_{l}=\frac{\left(\frac{1}{d^{l}}\right)}{u_{s}}$$

$$s_{x,y}=u_{1}p_{x,y}+\sum_{l=1}^{10}u_{l}p_{x,y,l}$$

These calculations were repeated for all wells containing at least one murine cell.

### Trajectory inference analysis

Genes expressed in fewer than five cells and cells expressing fewer than 100 genes were excluded. Variance stabilizing transformation was performed using the SCTransform (*48*) function in the R Seurat package. The resulting corrected count data in MSC in one tissue were used as the count matrix input in trajectory inference analysis, using the tradeSeq (*41*) package in R. Genes whose expression is associated with the proximity score were identified by the associationTest function in tradeSeq, based on a Wald test under the negative binomial generalized additive model. The *P* values were corrected using Benjamini-Hochberg multiple testing procedure, and genes with corrected *P* values smaller than 0.05 were considered to be significantly associated with the proximity score.

## SUPPLEMENTARY MATERIALS

Supplementary material for this article is available at http://advances.sciencemag.org/cgi/content/full/7/17/eabg4755/DC1

## Appendix

View/request a protocol for this paper from *Bio-protocol*.
